# Barrett's Esophagus in an Area with an Exceptionally Low Prevalence of *Helicobacter pylori* Infection

**DOI:** 10.5402/2011/394734

**Published:** 2011-06-26

**Authors:** Yeong Yeh Lee, Sharifah Emilia Tuan Sharif, Syed Hassan Syed Abd Aziz, S. Mahendra Raj

**Affiliations:** ^1^Department of Medicine, Universiti Sains Malaysia, Kubang Kerian, Kota Bahru, 16150 Kelantan, Malaysia; ^2^Department of Pathology, Universiti Sains Malaysia, Kubang Kerian, Kota Bahru, 16150 Kelantan, Malaysia; ^3^Department of Surgery, Universiti Sains Malaysia, Kubang Kerian, Kota Bahru, 16150 Kelantan, Malaysia; ^4^Department of Medicine, Pantai Hospital Kuala Lumpur, 59100 Kuala Lumpur, Malaysia

## Abstract

*Objective*. This study was undertaken to gain an insight into the relationship between *Helicobacter pylori* (*H. pylori*) infection, Barrett's esophagus and reflux esophagitis in an area of exceptionally low prevalence of *H. pylori* infection. *Methods*. A total of 1895 consecutive upper endoscopies performed between January 2005 and July 2007 were reviewed. 120 cases of columnar-lined esophagus and endoscopic esophagitis were evaluated. *H. pylori* infection was determined using the urease test and/or histology. *Results*. The rate of endoscopic esophagitis was 5.49% (80 Malays, 24 non-Malays) while histological reflux esophagitis was found in 3.75% (56 Malays, 15 non-Malays). Barrett's esophagus was present in 0.79% (11 Malays, 4 non-Malays). *H. pylori* infection was present in 8/120 or 6.67% subjects. *Conclusion*. The low rate of Barrett's esophagus in this population does not support the hypothesis that the absence of *H. pylori* infection is more than a minor risk factor for Barrett's esophagus.

## 1. Introduction

Reflux esophagitis is thought to be uncommon among Asians, but recent studies have shown that erosive esophagitis is more common than what was originally thought [1, 2]. Similarly, Barrett's esophagus, a precursor of esophagus adenocarcinoma, is thought to have a low prevalence among Asians. There are two reports from Peninsular Malaysia showing different rates of Barrett's esophagus in two different regions. Rajendra et al. [3] reported a 6% prevalence rate of Barrett's esophagus in the northwestern part of Peninsular Malaysia while Rosaida and Goh [4] in the Klang Valley of central Peninsular Malaysia reported a rate of 2%. The difference in rates may be related to differences in the ethnic composition of the population in these regions. The predominantly Malay population of northeastern Peninsular Malaysia has a naturally low *Helicobacter pylori (H. pylori) *prevalence rate of 4%-5%, a rate that is among the lowest reported in the world [5–7]. The *H. pylori *seroprevalence was also studied in the Aboriginal (Orang Asli) community from this region, and the prevalence rate was only 19% [8]. In a recent study, patients underwent upper endoscopy in northeastern Peninsular Malaysia; the rate of chronic atrophic gastritis was found to be 42%, and *H. pylori* infection was present in only 11% of the study sample [9]. The aim of this study was to determine the rate of Barrett's esophagus and histological oesophagitis in an endoscopy-based sample of this unique population with a view to gaining an insight into the relationship between *H. pylori* infection and Barrett's esophagus. This is especially pertinent in the light of recent suggestions of an inverse relationship between *H. pylori *infection, gastroesophageal reflux disease (GERD), Barrett's and esophageal adenocarcinoma.

## 2. Patients and Methods

A total of 1895 consecutive patients who underwent endoscopy for upper gastrointestinal symptoms between 2005 and 2007 at Hospital Universiti Sains Malaysia (USM) were recruited into the study. The upper gastrointestinal symptoms included dyspepsia (both typical and atypical), chest discomfort, epigastric pain, heartburn, acid regurgitation, dysphagia, nausea, and/or vomiting. This hospital is a teaching and tertiary referral centre serving Northeastern Peninsular Malaysia. All upper endoscopies (model GIF-140 and GIF-160; Olympus Medical Systems, Tokyo, Japan) during this period were performed by two experienced endoscopists (Y. Y. Lee, S. H. S. Abd Aziz) who initially agreed on a consensus of endoscopic assessment to reduce finding variations. If needed, patients were sedated accordingly. Patients with esophagitis visible on endoscopy and/or columnar-lined esophagus were biopsied. Patients aged from 18 years old and elder were included, and those with esophageal carcinoma, history of upper gastrointestinal surgery, esophageal varices, or repeated endoscopy were excluded. 

Endoscopic esophagitis was classified according to Los Angeles (LA) classification (Grade A to D) as described previously [10]. Barrett's esophagus was identified as an extension of columnar epithelium above the gastroesophageal junction. It was standard practice for all patients with esophagitis or suspected Barrett's esophagus to be biopsied at multiple levels at the squamocolumnar junction. *H. pylori* infection was routinely determined during endoscopy using the rapid urease test or CLO test (Ballard Medical Products, Utah, USA) and/or histology with or without Werthein stain from two tissue biopsies taken over the antrum and/or the body. 

Reflux esophagitis was defined histologically by the presence of basal cell thickening, elongation of papillae of the epithelium, dilated vessels in the papillae of lamina propria, and distended pale squamous cells. The severity of reflux esophagitis was recorded as mild, moderate, and severe based on the presence of lymphoplasma cells infiltration in the tissue sample, and a visual grading of severity was given by the pathologist based on the volume of cell infiltration [11]. Barrett's esophagus was confirmed histologically by the presence of intestinal metaplasia epithelium within the columnar-lined esophagus which contains goblet cells and stains positively with Alcian blue [12]. Dysplasia was identified by the presence of definite cytologic and architectural abnormalities in the neoplastic epithelium. Cases were excluded if the diagnosis of dysplasia was confounded by the presence of marked inflammation [13]. 

Statistical analysis was performed using SPSS software version 12.0.1 (SPSS Inc, Chicago, Ill, USA). Chi-square test or Fischer's exact test was used for categorical variables and independent *t* test for numerical variables where appropriate. A *P* value of less than 0.05 was considered statistically significant.

## 3. Results

The rate of esophagitis seen endoscopically was 5.49% (104/1895) of which 71 patients were later confirmed histologically (Table 1). More males had endoscopic esophagitis than females, but it was not statistically significant (*P* = 0.97).  Table 2 showed most patients had mild erosive esophagitis. *H. pylori* infection was present in six patients (6/104 or 5.77%) with endoscopic esophagitis and in two patients with columnar lined oesophagus (2/16 or 12.50%). The prevalence of Barrett's esophagus confirmed histologically was 0.78% (15/1895), while 0.84% (16/1895) had columnar lined esophagus. However, only 43.75% (7/16) of the patients suspected to have Barrett's esophagus on endoscopy fulfilled the histological criteria of Barrett's esophagus (Table 1). Of the patients with endoscopic esophagitis, 7.69% (8/104) had histologically confirmed Barrett's esophagus. In terms of ethnicity, non-Malays constituted 23.08% (24/104) of patients with endoscopic esophagitis and 26.67% (4/15) of patients with histologically confirmed Barrett's esophagus (Table 2). Compared to the demographics of the area and the ethnic distribution of all patients attending the hospital during study period, non-Malays were overrepresented among patients with reflux esophagitis and Barrett's esophagus. There was only one case of low-grade dysplasia seen, and the patient had background history of scleroderma. Normal histology was reported in 34 cases of which 31 cases were normal in erosive esophagitis seen endoscopically, and 3 cases were normal in suspected Barrett's esophagus.

## 4. Discussion

It is acknowledged at the outset that any prevalence study based on endoscopy is subject to selection bias. Observer variation among endoscopists in the recognition of esophagitis and Barrett's esophagus constitutes another limitation. Notwithstanding comparison with the data of other endoscoped series does permit useful inferences.

The 5.49% prevalence rate of endoscopic esophagitis in this study is generally lower than that reported in most other endoscopy-based studies especially those from the Western world (Table 3). In concordance with this observation, the prevalence of Barrett's esophagus is also low at 0.79%. This is perhaps not surprising as Barrett's esophagus is considered to be a consequence of long-standing gastroesophageal reflux disease. The differences in the prevalence rates of esophagitis and Barrett's esophagus reported in the other two studies from Malaysia may relate to differences in ethnic composition and socioeconomic demographics. Northeastern Peninsular Malaysia has a larger proportion of ethnic Malays (90%) than Northwestern (51.4%) or Central (50%) Peninsular Malaysia. Rajendra et al. showed that Indians are more predisposed to gastroesophageal reflux than Chinese or Malays demonstrating the potential role of ethnic differences [3]. This is supported by an observation by the same author of a predominance of human leucocyte antigen (HLA) B7 subtype among Indians with Barrett's [20]. Indeed even in our study non-Malays were overrepresented among patients with reflux esophagitis and Barrett's esophagus. 

There has been a suggestion that *H. pylori* infection rate is inversely related to the prevalence of GERD, Barrett's esophagus, and esophageal adenocarcinoma [21–23]. The low rate of esophagitis and Barrett's esophagus observed in this study despite the fact that *H. pylori* is almost absent does not support the notion that the absence of *H. pylori* infection plays more than a minor role as a risk factor for Barrett's esophagus and reflux esophagitis. Other factors relating to race, life style, and obesity are likely to be more important than *H. pylori*. The fact that non-Malays in this study were more likely to have esophagitis and Barrett's esophagus despite their well-documented higher prevalence of *H. pylori *infection also argues against the decline of *H. pylori* being an important factor in the etiopathogenesis of reflux disease. Indeed in northeastern region of Peninsular Malaysia, the age-standardised incidence of oesophageal adenocarcinoma a sequelae of Barrett's was found to be only 0.72/100,000 among the Malays [24]. Although progression from Barrett's to dysplasia is slow and rare (30% low-grade dysplasia and 5% high-grade dysplasia in six years) [25], it is notable that only one case of dysplasia was detected in this study, and even that was in a patient with scleroderma, a condition known to predispose to reflux esophagitis.

In conclusion, the low rate of Barrett's esophagus in this series from an area with an exceptionally low *H. pylori* prevalence does not support the hypothesis that the absence of *H. pylori* infection is more than a minor risk factor for Barrett's esophagus. This is corroborated by the observation that non-Malay subjects who have a higher prevalence of *H. pylori* were more likely to have Barrett's than the Malays.

## Figures and Tables

**Table 1:** Characteristics of study sample in relation to presence of esophagitis and Barrett's esophagus.

	Endoscopic esophagitis	Endoscopic Barrett's esophagus
Number of cases (*n*)	104	16
Age (Mean ± SD, years)	53.6 ± 1.5	61.4 ± 3.2
Gender		
Male (*n*)	59	9
Female (*n*)	45	7
Biopsy-confirmed cases (*n*)		
(1) Erosive esophagitis	65	6
(2) Barrett's esophagus without concomitant esophagitis	1	1
(3) Barrett's esophagus with concomitant esophagitis	7	6
Normal histology	31	3
Prevalence (%)	3.74	0.78
*Helicobacter pylori* (*n*)	6	2

SD: standard deviation.

**Table 2:** Ethnic distribution of subjects with reflux esophagitis and Barrett's esophagus.

	Malay	non-Malay
Endoscopically diagnosed	80	24
(1) Erosive oesophagitis (*n*)	46	14
LA grade A	13	4
LA grade B	14	4
LA grade C	7	2
LA grade D		
(2) Barrett's (*n*)	14	2
Biopsy-confirmed Barrett's (*n*)	11	4*
*Helicobacter pylori *positive	6	2^#^

LA: Los Angeles Classification for erosive oesophagitis; *N*: total number of upper endoscopies screened between 2005 to 2007. *3 Chinese, 1 Indian. ^#^1Chinese, 1 Indian.

**Table 3:** Reported prevalence of esophagitis and Barrett's esophagus in different populations based on endoscopy study.

	Region	Sample size (*n*)	Prevalence *n* (%) of	
			Esophagitis	Barrett's
Rajendra et al. [3]	Northwestern Peninsular Malaysia	1985	121 (6.1)	123 (6.2)
Rosaida and Goh [4]	Klang Valley, Peninsular Malaysia	1000	134 (13.4)	20 (2.0)
Current study	Northeastern Peninsular Malaysia	1895	104 (5.49)	15 (0.79)
Xiong et al. [14]	Guangzhou, China	2022	68 (3.4)	21 (1.0)
Wong et al. [15]	Hong Kong	16606	631 (3.8)	10 (0.06)
Yeh et al. [16]	Taiwan	464	66 (14.5)	9 (2.0)
Yilmaz et al. [17]	Turkey	18,766	2402 (12.8)	280 (1.5)
Ronkainen et al. [18]	Sweden	1000	155 (15.5)	16 (1.6)
Rex et al. [19]	USA	961	155 (16.1)	65 (6.8)
